# Supplementary material for: NRas Nanoclusters Mediate Crosstalk Between BRAF/ERK and PI3K/AKT Signaling in Melanoma Cells
Source: Int J Mol Sci. 2025 Dec 1;26(23):11647. doi: 10.3390/ijms262311647 (PMC12692423; doi:10.3390/ijms262311647)
Supplement: Supplementary file 1 [file ijms-26-11647-s001.zip › ijms-3876466-supplementary/ijms-3876466_Supplementary_Material.pdf]

Supplementary Material: NRas Nanoclusters Mediate Crosstalk Between BRAF/ERK and PI3K/AKT signalling in Melanoma Cells

Oren Yakovian [1], Julia Sajman [1,2] and Eilon Sherman [1,*]

[1] Racah Institute of Physics, The Hebrew University, Jerusalem 9190401, Israel; oren.yakovian@mail.huji.ac.il (O.Y.); julia.sajman@mail.huji.ac.il (J.S.)
[2] Walder Department of Bioinformatics, Tal Campus for Women, Jerusalem College of Technology, Havaad Haleumi 21, Givat Mordechai 91160, Jerusalem, Israel
* Correspondence: eilon.sherman@mail.huji.ac.il; Tel.: +972-2-6586878

Supplementary Figures and Legends

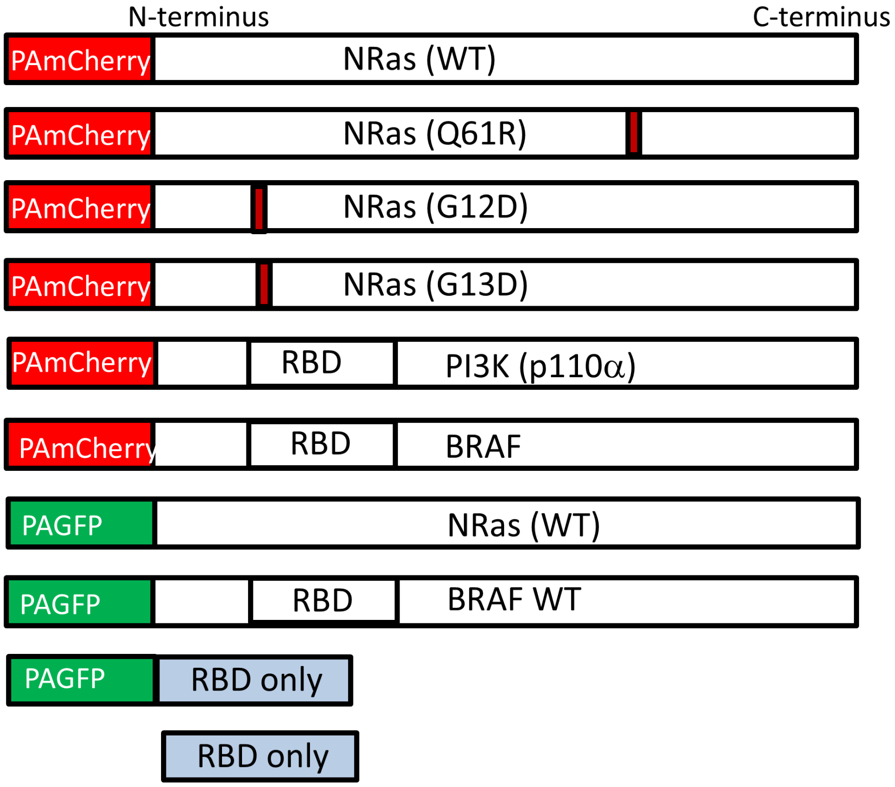

Figure S1. Constructs used for two-color PALM imaging.

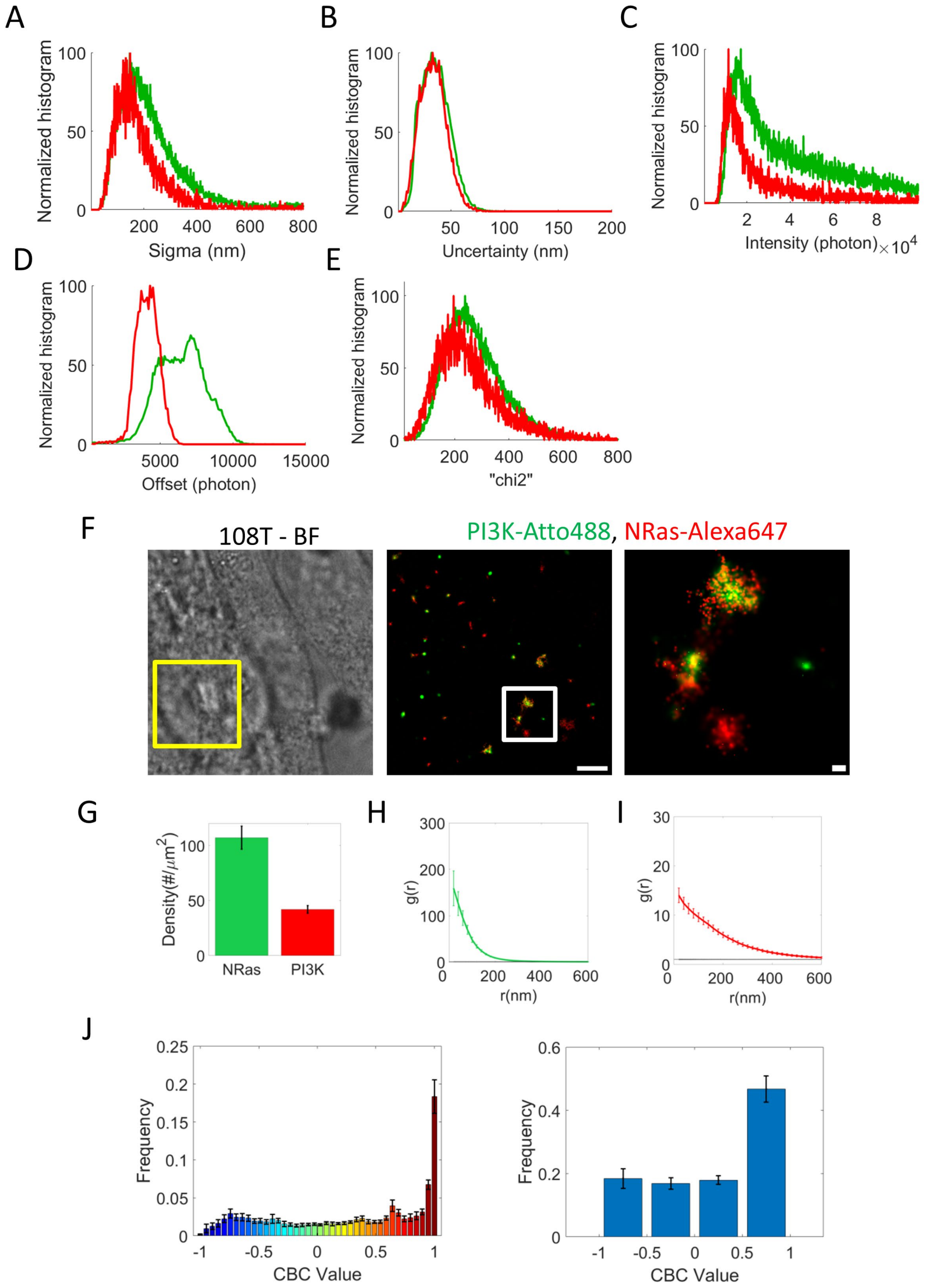

Figure S2. Performance analysis of fluorophores used for two-color PALM imaging and dSTORM imaging of NRas and PI3K clustering. **A-E.** Statistics on various parameters related to localizations using two-color PALM imaging, including Sigma (A), Uncertainty (B), Intensity (C), Offset (D) and Chi-squared (E). **F-J.** Direct STORM imaging of Nas and PI3K co-clustering at the plasma-

membrane of melanoma cells. **F.** Shown is a representative cell (N = 19). Zooms of left and middle images are shown on their right. Bars, 2 µm (middle) and 200 nm (right). **G.** The density of BRAF and NRas at the PM. **H,I.** PCF of NRas (H) and of PI3K (I). **J.** Coordinate-based colocalization (CBC) analysis of multiple cells (N=19). Right graph shows the distribution with coarse binning.

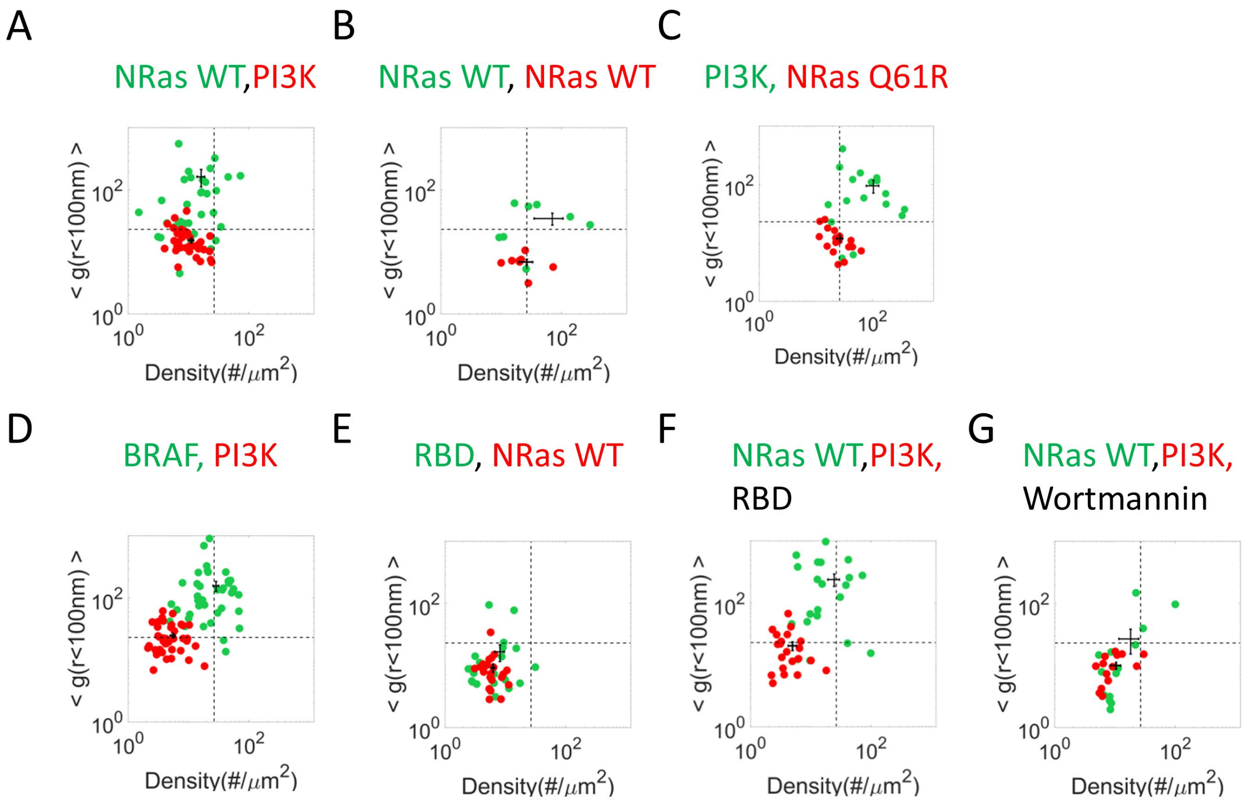

Figure S3. Density and self-clustering maps. **A-F.** Two-dimensional maps of self-clustering [value of g(0–100)] versus protein density. Values are shown for individual cells as discs, either in green or in red for the species shown. Note that both axes are logarithmic. Dashed black lines are guidelines for comparison of results with additional measurements. Shown are the results for: **A.** PAGFP-NRas-wt and PI3K (related to Fig. 1A-D), **B.** PAGFP-NRas-wt and PAmCherry-NRas-wt (related to Fig. 3A-D), **C.** PAGFP-PI3K and PAmCherry-NRas-Q61R (related to Fig. 3L-O), **D.** PAGFP-BRAF and PAmCherry-PI3K (related to Fig. 4A-D), **E.** PAGFP-RBD and PAmCherry-NRas-wt (related to Fig. 5A-D), **F.** PAGFP-NRas-wt and PAmCherry-PI3K, for cells treated with overexpressed RBD (related to Fig. 5F-I). **G.** PAGFP-NRas-wt and PAmCherry-PI3K, for cells treated with wortmannin (related to Fig. 5L-O).

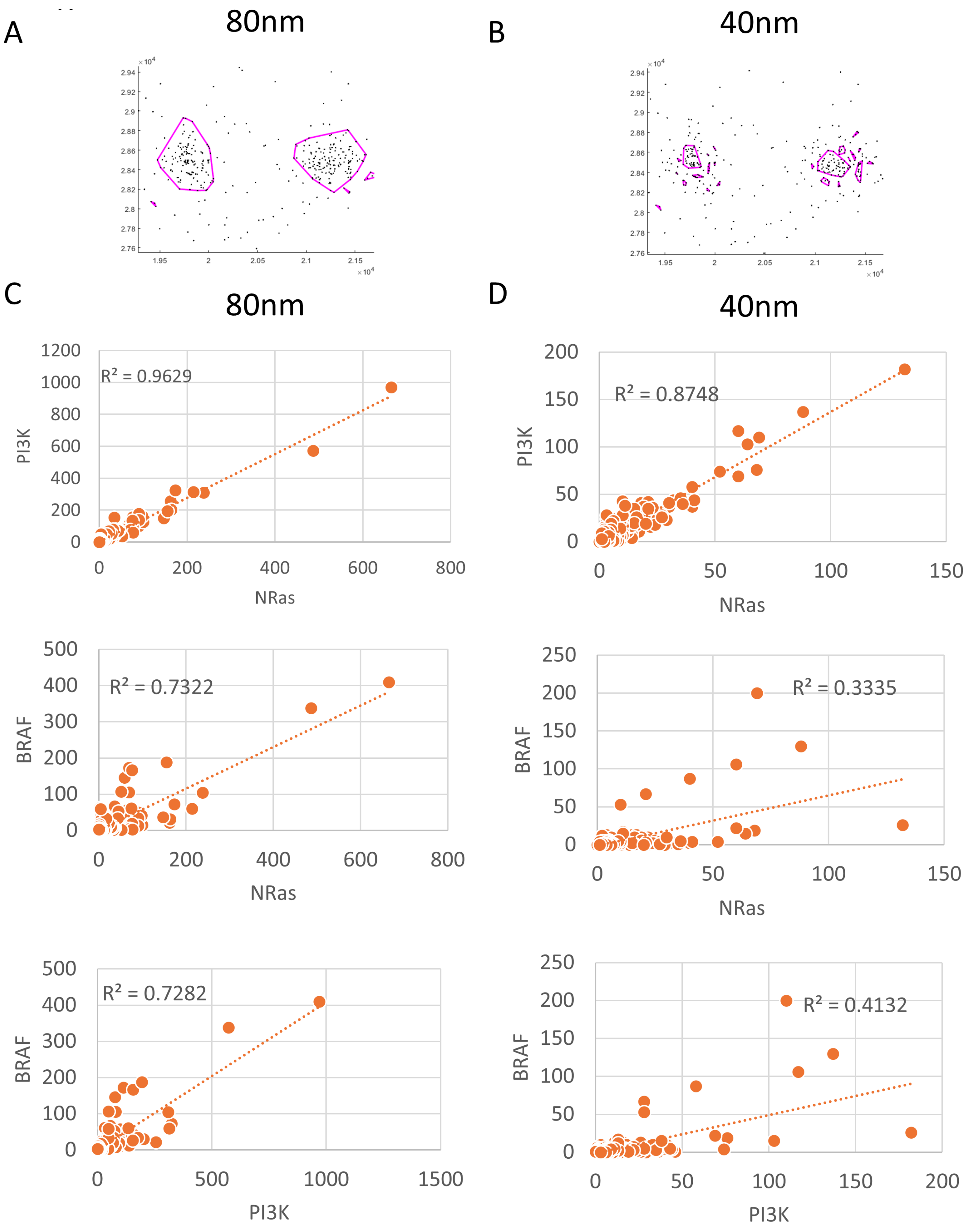

Figure S4. Co-clustering analysis of NRas, BRAF and PI3K in same clusters. **A,B.** Analysis of joint clustering of NRas, BRAF and PI3K (all shown as black dots). Clusters were identified using DBSCAN with a cutoff distance of either 80nm (A) or 40nm (B) between adjacent localizations. **C,D.** Co-clustering analysis of PI3K with NRas (top), BRAF and NRas (middle) and BRAF with PI3K (bottom) using the DBSCAN cutoff of 80nm (C) or 40nm (D).

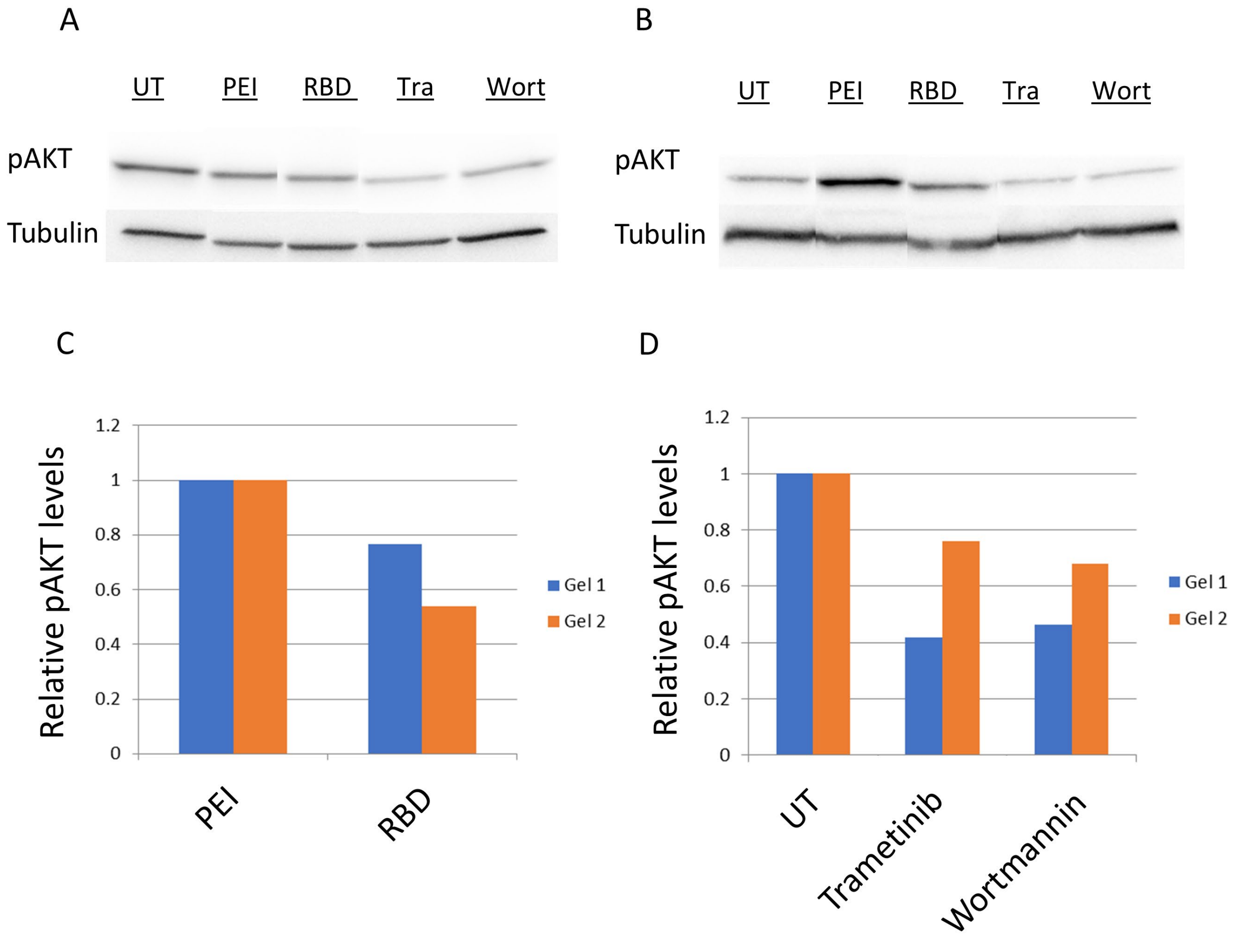

Figure S5. Inhibition of pAKT by RBD overexpression and small molecule drugs. **A,B.** Western blots of resting 108T melanoma cells. Wells are shown as follows: 'UT'- Untransfected cells, 'PEI' – Mocked transfected cells, 'RBD' – cell transfected with Flag-RBD vector, 'Tra' – cell treated with 10nM trametinib, 'Wort' – cell treated with 3nM wortmannin. Blots were stained pAKT. Shown are sections of two independent blots. **C,D.** Relative pAKT levels from the two blots under RBD over-expression (C) or under treatment with either trametinib or wortmannin (D).

Supplemental Tables

Table S1. Pairwise Pearson Correlation Coefficients.

80nm	647	561	Total
**647**	1	0.973428	0.979958
**561**	0.973428	1	0.976772
**488**	0.771712	0.746407	0.863737
**Total**	0.991648	0.979958	1

40nm	647	561	Total
**647**	1	0.914127	0.896435
**561**	0.914127	1	0.898584
**488**	0.533111	0.516123	0.827200
**Total**	0.896435	0.898584	1

Supplemental Movie

Movie M1. Dynamics of NRas and PI3K co-clustering at the plasma-membrane of melanoma cells. (Top left). Two-color PALM (TIRF) imaging of live 108T melanoma cells expressing PAGFP-PI3K (green) and PAmCherry-NRas (red). The cells were dropped on coated with PLL and EGF under the microscope and imaged from contact identification. A representative cell (n=9) is shown at different timepoints along imaging. (Top right). CBC analysis of the effective frames of the imaged cell in panel A. PI3K proteins are colored by their CBC value (i.e. their proximity to NRas). In the left and right panels, filled and empty arrowheads exemplify clusters with persistent or transient NRas-PI3K interactions, respectively. (Bottom left). The distribution of CBC values of PI3K. (Bottom right) The CBC ratio over time.
